# Limited polymorphisms in *k13* gene in *Plasmodium falciparum* isolates from Dakar, Senegal in 2012–2013

**DOI:** 10.1186/1475-2875-13-472

**Published:** 2014-12-04

**Authors:** Marylin Torrentino-Madamet, Bécaye Fall, Nicolas Benoit, Cheikhou Camara, Rémy Amalvict, Mansour Fall, Pierre Dionne, Kadidiatou Ba Fall, Aminata Nakoulima, Bakary Diatta, Yaya Diemé, Didier Ménard, Boubacar Wade, Bruno Pradines

**Affiliations:** Equipe Résidente de Recherche en Infectiologie Tropicale, Institut de Recherche Biomédicale des Armées, Hôpital d’Instruction des Armées, Marseille, France; Unité de Recherche sur les Maladies Infectieuses et Tropicales Emergentes, UM 63, CNRS 7278, IRD 198, Inserm 1095, Aix Marseille Université, Marseille, France; Centre National de Référence du Paludisme, Marseille, France; Laboratoire d’Etude de la Chimiosensibilité du Paludisme, Fédération des Laboratoires, Hôpital Principal de Dakar, Dakar, Sénégal; Service des Urgences, Hôpital Principal de Dakar, Dakar, Sénégal; Service de réanimation Médicale, Hôpital Principal de Dakar, Dakar, Sénégal; Maternité Hôpital Principal de Dakar, Dakar, Sénégal; Service de Pathologie Infectieuse, Hôpital Principal de Dakar, Dakar, Sénégal; Service de Pédiatrie, Hôpital Principal de Dakar, Dakar, Sénégal; Malaria Molecular Epidemiology Unit, Institut Pasteur du Cambodge, Phnom Penh, Cambodia; Chefferie, Hôpital Principal de Dakar, Dakar, Sénégal; Unité de Parasitologie et d’Entomologie, Département des Maladies Infectieuses, Institut de Recherche Biomédicale des Armées, Brétigny sur Orge, Bretigny sur Orge, France

**Keywords:** Malaria, *Plasmodium falciparum*, Anti-malarial drug, Resistance, Molecular marker, artemisinin, K13-propeller, Senegal

## Abstract

**Background:**

The emergence of *Plasmodium falciparum* resistance to artemisinin and its derivatives, manifested as delayed parasite clearance following the treatment, has developed in Southeast Asia. The spread of resistance to artemisinin from Asia to Africa may be catastrophic for malaria control and elimination worldwide. Recently, mutations in the propeller domain of the *Kelch 13* (*k13*) gene (*PF3D71343700)* were associated with *in vitro* resistance to artemisinin and with delayed clearance after artemisinin treatment in southern Asia. The aim of the study was to characterize the genetic variability of *k13* and to evaluate the molecular resistance to artemisinin for the first time in Senegal.

**Methods:**

*Plasmodium falciparum* isolates were collected from 138 malaria patients in Dakar and its districts during the rainy season of October 2012 to January 2013 at the Hôpital Principal de Dakar. The *k13* gene was amplified using nested PCR and sequenced.

**Results:**

A very limited variability within the *k13* gene in Senegalese *P. falciparum* isolates was identified. No polymorphism was detected in the six *k13*-propeller blades. Only two mutations, T149S (6.3%) and K189T (42.2%), and one (N) or two (NN) asparagine insertion at the codon 142 (4.7 and 6.3%, respectively) were detected in the *Plasmodium*/Apicomplexa-specific domain. None of the polymorphisms associated with artemisinin resistance in Southeast Asia was detected in the 138 *P. falciparum* from Dakar.

**Discussion:**

The present data do not suggest widespread artemisinin resistance in Dakar in 2012–2013. Notably, the C580Y, R539T or Y493H substitutions that were associated with *in vitro* resistance or delayed parasite clearance in Southeast Asia were not observed in Dakar, nor were any of the polymorphisms observed in parasites from Southeast Asia, nor the M476I mutation that was selected *in vitro* with artemisinin pressure in a African parasite line.

## Background

Malaria is transmitted in Dakar and its surrounding suburbs with a spatial heterogeneity of the human biting rate, which ranged from 0.1 to 250 bites per person per night during the rainy season from 2007 to 2010 [1, 2]. In 2008, the *Plasmodium falciparum* prevalence varied from 0.9 to 7.4% in asymptomatic women and children in Dakar [3]. Morbidity in public health facilities decreased from 17.9% in 2007 to 2.6% in 2008 in Dakar [4].

In 2006, the Senegalese National Malaria Control Programme recommended artemisinin-based combination therapy (ACT) as the first-line treatment for uncomplicated malaria. The combination sulphadoxine-pyrimethamine and amodiaquine treatment was changed to artemether-lumefantrine and artesunate-amodiaquine. Since 2006, more than 1.5 million ACT has been administered in Senegal [5]. In 2006, the Senegalese National Malaria Control Programme also recommended testing for all suspected cases of malaria with the *P. falciparum* histidine-rich protein 2-based rapid diagnostic test. Since this time, ACT use has been restricted to confirmed malaria cases to reduce drug pressure. In 2009, 184,170 doses of ACT were dispensed in Senegal [6]. In addition, the National Malaria Control Programme also recommended the use of SP as an intermittent preventive treatment (IPT), now named seasonal malaria chemoprevention (SMC), during pregnancy and for children. However, the single use of SP as seasonal IPT is inadvisable; for instance, SP must be used in combination with amodiaquine, artesunate or piperaquine. Seasonal IPT with SP and one dose of artesunate resulted in a 90% reduction in the incidence of clinical malaria in Senegal [7, 8].

However, the emergence of *P. falciparum* resistance to artemisinin and its derivatives, manifested as delayed parasite clearance following the treatment with artesunate monotherapy or ACT, has developed in Southeast Asia [9–12]. The spread of artemisinin resistance from the Greater Mekong subregion to Africa may be catastrophic for malaria control and elimination around the world. The spread of anti-malarial drug resistance from Southeast Asia to Africa has previously happened with chloroquine and SP [13, 14]. Vigilant surveillance for resistant parasites is warranted.

Recently, mutations in the propeller domain of the *kelch 13* (*k13*) gene were associated with *in vitro* resistance to artemisinin and with delayed clearance after artemisinin treatment in Southeast Asia [12, 15–17]. The aim of the study was to characterize the variability of *k13* gene for the first time in Senegal.

## Methods

### Patient and sample collection

*Plasmodium falciparum* isolates were obtained from patients diagnosed with malaria, who live in Dakar and its districts (>90%) and did not travel during the previous month, during the rainy season of October 2012 to January 2013 (138 patients, 37% female). The patients with malaria were recruited at the Hôpital Principal de Dakar, a military hospital. Venous blood samples were collected in Vacutainer® ACD tubes (Becton Dickinson, Rutherford, NJ, USA) prior to patient treatment. Of the 138 patients, 57% were recruited from the emergency department and other patients were recruited from the intensive care unit (20%), maternity department (7%), infectious diseases department (5%), paediatric department (3%) and other units (8%). Information on anti-malarial treatment prior to admission was not available. Informed verbal consent from the patients and/or their parents/guardians was obtained before blood collection; the study was approved by the ethical committee of the Hôpital Principal de Dakar. Thin blood smears were stained using a RAL® kit (Réactifs RAL, Paris, France) and were examined to determine *P. falciparum* density and to confirm monoinfection by *P. falciparum*.

Evaluation of *k13*-propeller polymorphisms was performed using the same venous blood sample used for this diagnostic analysis. The patients were successfully treated with quinine (98%) and artemether-lumefantrine (2%).

### Analysis of *Plasmodium falciparum*isolates

The total genomic DNA of each isolate was extracted using the QIAamp® DNA Mini kit according to the manufacturer’s recommendations (Qiagen, Germany). The *k13*-propeller gene was amplified by PCR using the following primers: for the primary PCR (K13_PCR_F 5’-GGGAATCTGGTGGTAACAGC-3’ and K13_PCR_R 5’-CGGAGTGACCAAATCTGGGA-3’) and for the nested PCR (K13_N1_F 5’-GCCTTGTTGAAAGAAGCAGA-3’ and K13_N1_R 5’-GCCAAGCTGCCATTCATTTG-3’) [15]. For primary PCR, 5 μL of genomic DNA were amplified with 1X final of reaction buffer (Eurogentec, Belgium), 200 μM of a deoxynucleoside triphosphate mixture (dGTP, dATP, dTTP and dCTP) (Eurogentec, Belgium), 2.5 mM MgCl_2_, 250 nM of each primer and 1.25 U Red Diamond Taq® polymerase (Eurogentec, Belgium) to a final volume of 25 μl. The thermal cycler (T3 Biometra, Archamps, France) was programmed as follows: 15 min at 95°C, then 30 cycles of 30 sec at 95°C, 2 min at 58°C, 2 min at 72°C and final extension 10 min at 72°C. For the nested PCR, 5 μL of primary PCR amplicons were amplified under the same conditions with two U Red Diamond Taq® polymerase (Eurogentec, Belgium) to a final volume of 50 μl. The PCR thermocycling conditions were: 15 min at 95°C, then 40 cycles of 30 sec at 95°C, 1 min at 60°C, 1 min at 72°C and final extension 10 min at 72°C.

The sequencing reaction contained 4 μl of BigDye Terminator® v3.1 mix (Life Technologies, CA, USA), 0.8 μM of primer described above, and 4 μL PCR amplicon in a total of 20 μl volume. The cycle conditions were initial denaturation at 96°C for 5 min followed by 30 cycles at 96°C for 10 sec, 50°C for 5 sec, and 60°C for 4 min. Excess dye terminators were removed with a BigDye XTerminator® Purification Kit (Life Technologies, CA, USA). The samples were loaded on an ABI Prism 3100 analyzer (Applied Biosystems) according to the manufacturers’ instructions. The sequences were analysed using Vector NTI advance (TM) software (version 11, Invitrogen, Cergy Pontoise, France) to identify specific single nucleotide polymorphism (SNP) combinations.

## Results

The entire *k13* gene was successfully sequenced in 64/138 *P. falciparum* isolates. The propeller domain gene was successfully in the 138 malaria episodes diagnosed from October 2012 to January 2013. Compared to the reference 3D7 strain (PF3D7_1343700 reference coding sequence), the SNPs observed were shown in Table 1. The polymorphisms T149S (6.3%) and K189T (42.2%) were not found in same isolates. The K189T mutation was found in two out of the three isolates with one asparagine (N) insertion (NN at codon 142). Notably, the C580Y, R539T or Y493H substitutions that were associated with *in vitro* resistance or delayed parasite clearance in Southeast Asia were not observed in Dakar, nor were any of the polymorphisms observed in parasites from Southeast Asia, nor the M476I mutation that was selected *in vitro* with artemisinin pressure.

**Table 1:** **Polymorphisms observed in the** ***K13*** **gene in 138** ***Plasmodium falciparum*** **isolates from Dakar, Senegal collected from October 2012 to January 2013**

Codon position	Amino acid reference	Amino acid mutation or asparagine insertion	n/N (%)
142	N	NN	3/64 (4.7%)
142	N	NNN	4/64 (6.3%)
149	T	S	4/64 (4.7%)
189	K	T	27/64 (42.2%)

All data are relative to the PF3D7_1343700 reference coding sequence.

n = number of samples with mutant allele or insertion.

N = number of samples sequenced at locus.

## Discussion

The emergence of *P. falciparum* resistance to artemisinin and its derivatives, manifested as delayed parasite clearance following the treatment with artesunate monotherapy or ACT, has recently developed in Southeast Asia [9, 12]. This clinical resistance was correlated with *in vitro* resistance, manifested by an increase in the ring-stage survival rate after contact with artemisinin [18, 19]. Mutations in the *P. falciparum k13* gene that encodes the kelch propeller domain were associated with *in vitro* resistance to artemisinin and with delayed clearance after artemisinin treatment in Southeast Asia [12, 15–17]. These works reported an *in vitro* artemisinin resistance associated with the M476I mutation in *k13*-propeller gene in African genetic background parasite (F32 Tanzania) that was selected *in vitro* with artemisinin pressure, a prolonged parasite *ex vivo* survival associated with the Y493H, I543T, R539T, and C580Y mutations, and an *in vivo* delayed parasite clearance associated with the Y493H, R539T and C580Y mutations. In addition, a recent report supports the causal role of *k13*-propeller mutations in conferring resistance to artemisinin, and specially the role of the C580Y mutation by genome manipulation [20]. The mutation C580Y was introduced in the genome of a *P. falciparum* strain susceptible to artemisinin using the CRISPR-Cas9 system, and consequently increased the ring-stage parasite survival in presence of artemisinin. However, the presence of multiple, population-specific mutations responsible for artemisinin resistance leads to independent emergence of resistance in multiples geographic locations in Southeast Asia [12, 17].

None of the polymorphisms associated with artemisinin resistance in Southeast Asia were detected in the 138 *P. falciparum* isolates from Dakar. A very limited variability was identified within the *k13* gene in Senegalese *P. falciparum* isolates. No polymorphism was detected in the six *k13*-propeller blades. Only two mutations, T149S and K189T, and an insertion of N or NN at codon 142 were detected in the *Plasmodium*/Apicomplexa-specific domain. The K189T mutation was present in 42.2% of the samples. This mutation was previously found in 34.4% (10/29) of the isolates tested in Uganda [21] and in one isolate in Bangladesh [17]. Among the 20 isolates with K189T mutation (unknown location of collection) previously found, all except one were associated with parasite clearance half-life <5 hours [12]. This cut-off of 5 hours was determined on the basis of 90^th^ centile for parasite clearance half-life in 2004 when resistance had just emerged in the Thailand-Myanmar border. However, parasites clearance rates are still influenced by many factors, including pharmacokinetics of anti-malarial drugs as well as host-immunity. A shorter cut-off needs to be considered for studies in settings of high transmission where patient immunity might be at a higher level. In Bangladesh, the only one isolate with K189T mutation was associated with parasite clearance half-life >5 hours [17]. The isolates with T149S mutation (unknown location of collection) previously found were associated with parasite clearance half-life <5 hours [12]. The role of these two mutations is not well established although these mutations seem to be associated with parasite clearance half-life <5 hours. Further studies are needed to better characterize these variations. However, the absence of putative *P. falciparum* artemisinin resistance mutations in Senegal is consistent with the efficacy of ACT in Senegal [22, 23].

The polymorphisms associated with artemisinin resistance in Southeast Asia were not detected in other countries in Africa with the exception of the P553L which was detected in one isolate in Mali [12, 23, 24]. In Uganda, the prevalence of *k13*-propeller polymorphisms was not associated with the persistence of parasites after two days following treatment with artemether-lumefantrine [21]. However, due to the high baseline parasitaemia in Uganda, persistent parasitaemia two days after the onset of therapy is likely not a reliable indicator of resistance in Uganda. When artemisinin resistance will emerge in Africa, it may be due by the spread of resistant parasites imported from Southeast Asia and/or by selecting for *de novo* evolution of resistance (uncommon mechanisms between Asia and Africa).

The V520A mutation that was identified in 13 of 14 parasites populations from West Africa (Gambia, Mali, Ghana, Burkina Faso), Central Africa (Democratic Republic of Congo) and East Africa (Kenya, Tanzania, Malawi) was not detected in Dakar [23]. This mutation was not found in Uganda [24].

These results are encouraging and suggest that artemisinin resistance is not yet established in Senegal. None of the mutations that are associated with artemisinin resistance in Southeast Asia are found in Africa [21, 24]. However, numerous novel *k13* propeller coding polymorphisms circulate in Africa. The phenotypes of these coding substitutions are unknown and will require further characterization to better characterize the clinical impact in artemisinin resistance in Africa.
